# Development and performance evaluation of sustainable false banana fiber reinforced composite fan blades

**DOI:** 10.1038/s41598-026-42862-9

**Published:** 2026-03-14

**Authors:** Yerdawu Zeleke Gebremaryam, Haile Simachew, Worku Tegegne Molla, Sivasubramanian Palanisamy, Saleh A. Alfarraj, Sulaiman Ali Alharbi, Mohamed Abbas, Shaeen Kalathil, Mezigebu Belay

**Affiliations:** 1 Center of Chemical Engineering, ITMO University, St. Petersburg, Russia; 2 Department of Mechanical Engineering, Wolkite University, Wolkite, Ethiopia; 3 Textile Engineering and Materials Research Group (TEAM), De Montfort University, Leicester, UK; 4 Department of Mechanical Engineering, School of Engineering, Mohan Babu University, Tirupati, Andhra Pradesh 517102 India; 5 Department of Zoology, College of Science, King Saud University, P.O. Box 2455, 11451 Riyadh, Saudi Arabia; 6 Department of Botany and Microbiology, College of Science, King Saud University, P. O. Box.2455, 11451 Riyadh, Saudi Arabia; 7 Electrical Engineering Department, College of Engineering, King Khalid University, 61421 Abha, Saudi Arabia; 8 Department of Condensed Matter Physics, Saveetha School of Engineering, Saveetha Institute of Medical and Technical Sciences, SIMATS, Chennai, India; 9 Department of Electrical Engineering, College of Engineering, Princess Nourah bint Abdulrahman University, P.O. Box 84428, 11671 Riyadh, Saudi Arabia; 10 Department of Metallurgical and Materials Engineering, College of Engineering, Ethiopian Defence University, 1041 Bishoftu, Ethiopia

**Keywords:** False banana fiber, Unsaturated polyester, Ceiling fan blade, Physico-mechanical properties, Finite element analysis, Engineering, Materials science

## Abstract

The use of textile fibre-reinforced composite materials for many alternative applications has significantly increased in the twenty-first century due to their lightweight nature and high strength-to-weight ratio. In this study, false banana fibres were used as reinforcement and unsaturated polyester resin as the matrix. The optimal ratio of fibre to matrix was established through an analysis of physico-mechanical parameters, including tensile, compressive, and flexural strengths, water absorption, and void fraction, utilising Design Expert software. Additionally, deformation, Von Mises stress, Von Mises strain, and velocity were analyzed using ANSYS simulation software. The composite exhibited water absorption of 1.5% over 24 to 48 h, a void fraction of 1.02%, a tensile strength of 33.15 MPa, a compressive strength of 29.69 MPa, and a bending or flexural strength of 28.85 MPa. Furthermore, the ANSYS results showed a maximum deformation of 0.60887 mm, a maximum equivalent elastic strain of 0.0018815, a minimum value of 1.0375 × 10^–10^, a maximum equivalent stress of 22.27 MPa, a minimum of 1.3877 × 10^–5^ MPa, and a velocity streamline of 14.97 m/s at 21 rad/s. The simulated stresses were well below the material’s measured strength limits, indicating a safe design under the analysed conditions. The weight of the developed composite blade was 31% lower than that of a conventional aluminum blade.

## Introduction

The ceiling fan is one of the most important electrical devices, and is a relatively low-power-consumption device in most industrial and commercial applications^1^. Ceiling fans are commonly used in warm regions to maintain indoor comfort, both for personal use and in meeting spaces. They are also employed in public areas, conference rooms, and other workplaces to ensure comfort^2^. Ceiling fans are mounted on the room’s ceiling to create a cooling effect through air circulation^3^. Significant efforts have been made to improve energy efficiency and reduce electrical consumption by optimizing the management of available resources and minimizing ceiling fan blade weight^4^. Existing aluminium fan blades offer advantages such as lower density compared to steel and corrosion resistance; however, they also have notable drawbacks, including a low strength-to-weight ratio and issues with paint and coating adhesion. To overcome these limitations, there has been a shift toward using fiber-reinforced composite materials for fan blades.

A composite material is composed of two or more distinct materials joined together^5^. It consists primarily of a matrix, which forms the continuous phase, and a secondary phase, usually the reinforcement, which is typically discontinuous^6^. The performance of composite materials largely depends on their constituent components and the production methods employed. To identify the optimal material for a specific application, it is essential to investigate the functional properties of all available fibers, their classifications, and the manufacturing techniques used in composite production^7^.

Natural fibers possess significant potential for reinforcing composite materials due to their lightweight nature, cost-effectiveness, high strength-to-weight ratio, biodegradability, recyclability, corrosion resistance, and attractive aesthetic qualities^8^. Additionally, their non-toxicity ensures minimal environmental impact^9^. Natural fiber-reinforced composites provide both cost savings and weight reduction^10^. However, two main issues must be addressed to effectively utilize composites reinforced with natural fibers: water absorption and resin compatibility^11,12^. The effectiveness of natural fibers such as hemp, kenaf, and bamboo has been evaluated in composites with an alkali-activated metakaolin^13^. Natural fibers have attracted significant attention from researchers and industry due to their environmental friendliness and contribution to sustainable practices. To better balance ecological, social, and economic factors, several companies have begun implementing eco-friendly technologies^14^. The increasing demand for sustainable materials and growing environmental awareness have led to a surge in the use of natural fibers as reinforcements or fillers. It has been demonstrated that incorporating natural fibers into thermoplastic matrices significantly enhances their mechanical properties, particularly stiffness and rigidity.

Recent research trends have shown an increasing reliance on finite element modeling (FEM) and simulation to assess the mechanical behavior of synthetic and natural fiber-reinforced composites, particularly in structural and rotating component applications. For example, FEM has been extensively used to predict stress distribution, deformation, and failure modes of synthetic composite rotor blades and fan components^15^. Similarly, natural fiber composites (e.g., jute, flax, and banana fiber) have been simulated using FEM to evaluate their potential for lightweight structural components with competitive performance, provided the fiber orientations and matrix composition are optimized^16^**.** These simulation-based studies provide valuable information for optimal design, damage prediction, and performance assessment under various loading conditions, contributing to the development of sustainable composite structures.

Natural fibers include wood, sisal, hemp, coconut, flax, jute, abaca, banana leaf fibers, bamboo, and other fibrous materials, which are used as reinforcements due to their good mechanical strength. The main chemical components that determine the physical and mechanical properties of these fibers are cellulose, hemicellulose, and lignin. The chemical composition of false banana fiber consists of 64.46% cellulose, 22.47% hemicellulose, 6.88% acid-insoluble lignin, 5.66% ash, and 0.54% solvent extractives. Additionally, the physical and mechanical properties of false banana fiber are characterized by diameter, linear density, moisture content, and elongation at break. Specifically, false banana fiber has a diameter of 128 μm, a linear density of 8.8 tex, a tensile strength of 352 MPa, and an elongation at break of 3.2%. The excellent tensile strength of false banana fiber is attributed to its high cellulose content and favorable crystallite orientation. These properties make false banana fiber a promising candidate for natural fiber–reinforced biocomposite production^17^.

Moreover, blending resins such as polyethylene, polypropylene, and PVA with natural fibers like pineapple, bamboo, jute, sisal, and banana enhances their mechanical properties and increases thermal stability^18,19^. Natural fibers can absorb moisture, which can significantly affect the mechanical properties of the composite due to challenges in interfacial bonding between the matrix and the fibers^20^. Thermoplastics and thermosetting polymers are the two main types of polymers used as matrices in composites. Thermosetting polymers, due to their extremely low viscosity, can be added to fibers at low pressure, making them easy to process and widely used in structural composite materials^21,22^. Therefore, common methods for developing composite materials include hand lay-up, resin injection, compression molding, pultrusion, and filament winding^23^. This research employs universal software packages for numerical simulation of continuum mechanics, specifically ANSYS Mechanical and ANSYS CFD, to analyze the responses of ceiling fan blades under specified loading conditions, including deformation, von Mises stress, and strain. Numerical simulation and finite element analysis involve advanced analytical techniques, the selection of suitable and accurate methods, and the use of software packages to simulate external loads^24^. The computational fluid dynamics (CFD) model is particularly effective, as it can predict airflow patterns and velocity distributions in structures, and is both practical and cost-effective^25^.

Commonly, ceiling fan blades are made from aluminum or synthetic fibers such as glass. In the present work, a ceiling fan blade was developed using a natural fiber (false banana fiber)–reinforced composite, consolidated with a thermosetting polymer (unsaturated polyester resin) as the matrix. Its physico-mechanical properties were analyzed, and a finite element analysis of the ceiling fan blade was conducted. Furthermore, the weight of the fan blade was compared with that of conventional aluminum fans, as a higher weight can reduce efficiency, affect fan performance, require more energy to rotate, and potentially increase energy consumption.

## Materials and methods

### Materials

The materials used in this research were alkali-treated (NaOH) false banana fiber, unsaturated polyester resin (LY556), and hardener (HY951), supplied by Herenba Instruments and Engineers.

### Method

#### Alkaline treatment of false banana fibers

**Fig. 1:**
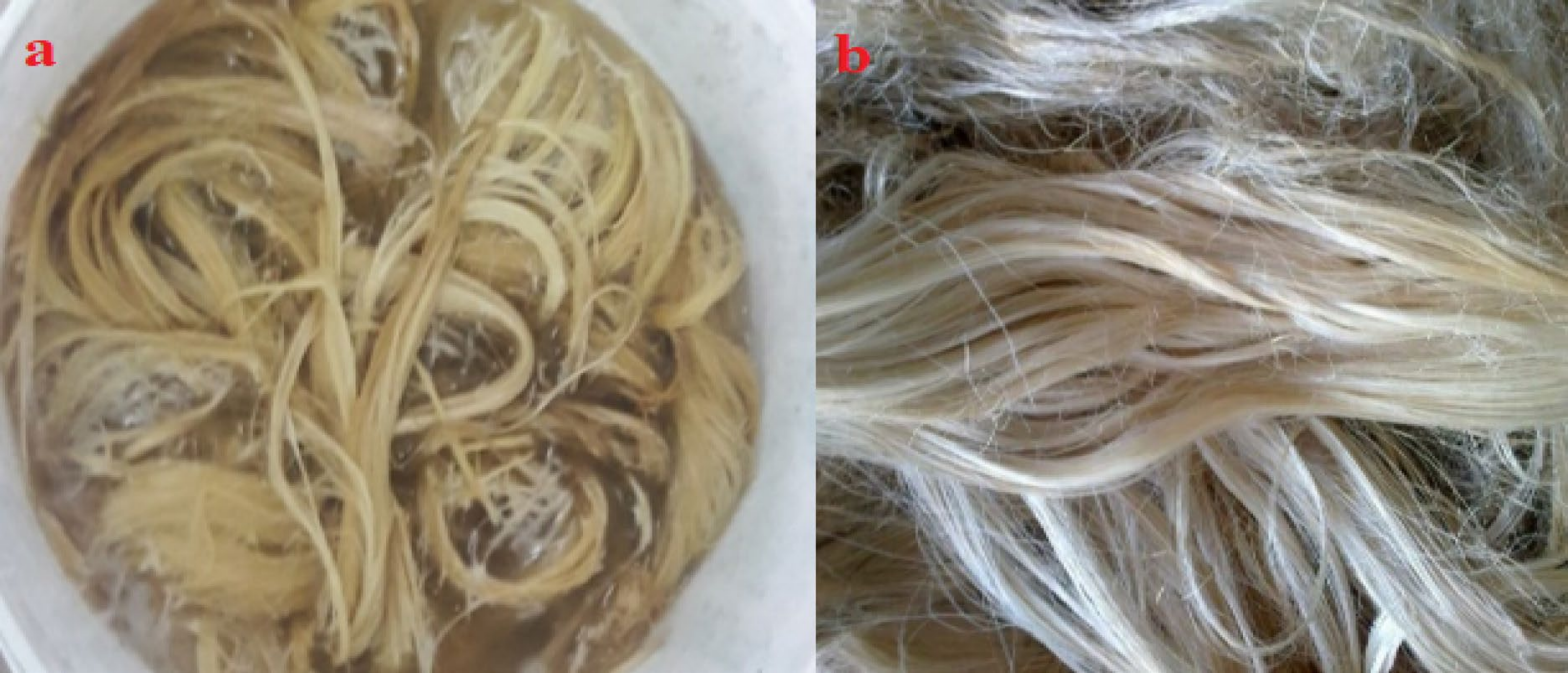
Alkaline (NaOH) treatment of false banana fiber (**a**) Treated; (**b**) Dried after treatment.

Natural fiber composites have drawbacks, including variations in properties such as poor matrix compatibility, high moisture absorption, and lower thermal stability. To address these issues, natural fibers are subjected to surface modifications, such as alkali treatments. Alkaline treatment of natural fibers enhances mechanical and morphological properties, including tensile and flexural strength, while reducing water absorption. Thus, alkaline treatment of false banana fiber was performed to improve inadequate adhesion between the fiber and matrix; both physical and chemical treatments are recommended for fiber surface modification. The false banana fiber was treated with sodium hydroxide (NaOH) before being mixed with unsaturated polyester resin to remove waxes, lignin, and hemicellulose, which increases surface roughness and improves adhesion with the polyester matrix^26^. The alkali solution concentrations used were 2.5%, 5%, and 10%, based on the material-to-liquor ratio (MLR) of false banana fibers soaked for 30 min. To remove excess NaOH, the fibers were washed with distilled water and then dried at 30 °C for 48 h^27,28^. Figure 1 illustrates the false banana fiber treatment employed in this study.

**Fig. 2:**
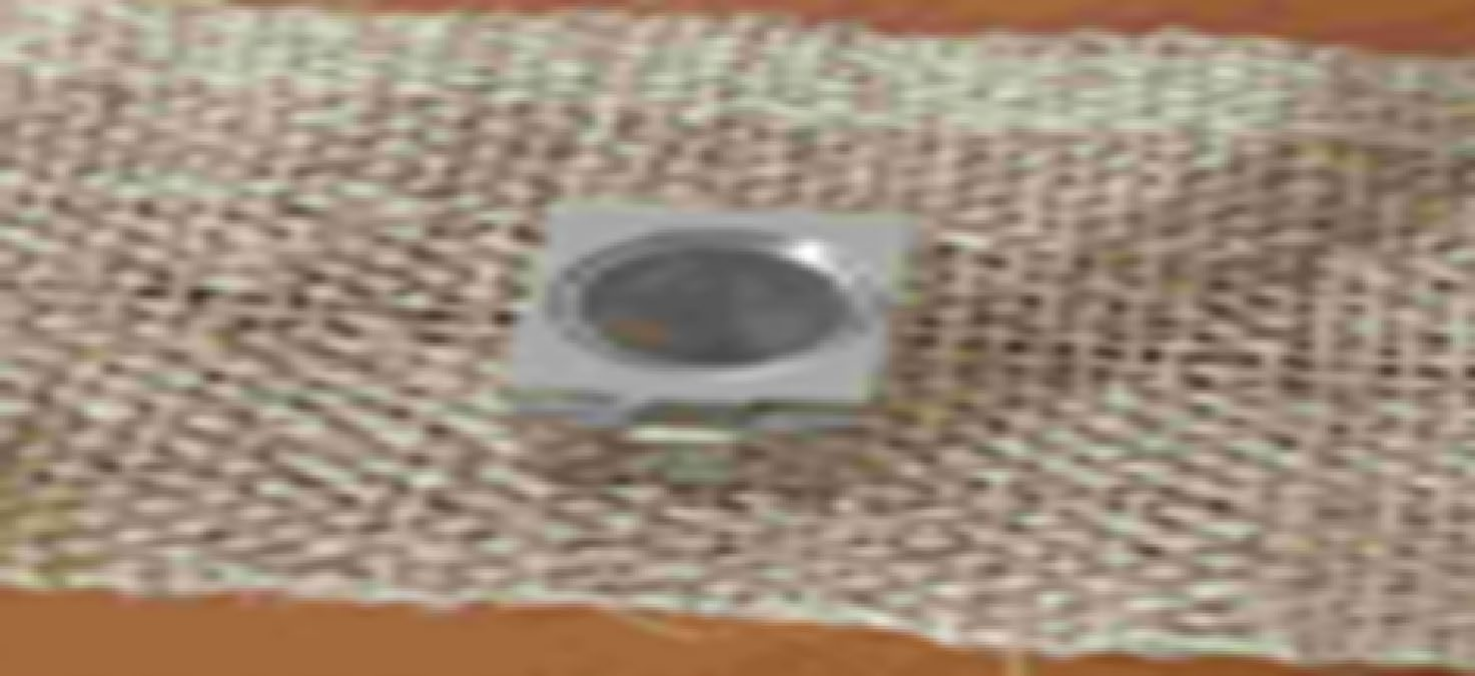
Number of picks and ends determination.

In this study, plain mat weave was used as a reinforcement material. The number of threads in warp and weft directions of plain mat weave, and the weight of mat weaves were determined as shown in Fig. 2.

The average result of five different places of the developed sample is shown in Table 1.

**Table 1:** Plain mat weave thread density.

For warp	Number of ends
Ends per ½ inch	5
Number of ends per inch (EPI)	10

For weft	Number of picks
Number of picks per ½ inch	4
Number of picks per inch (PPI)	9

**Fig. 3:**
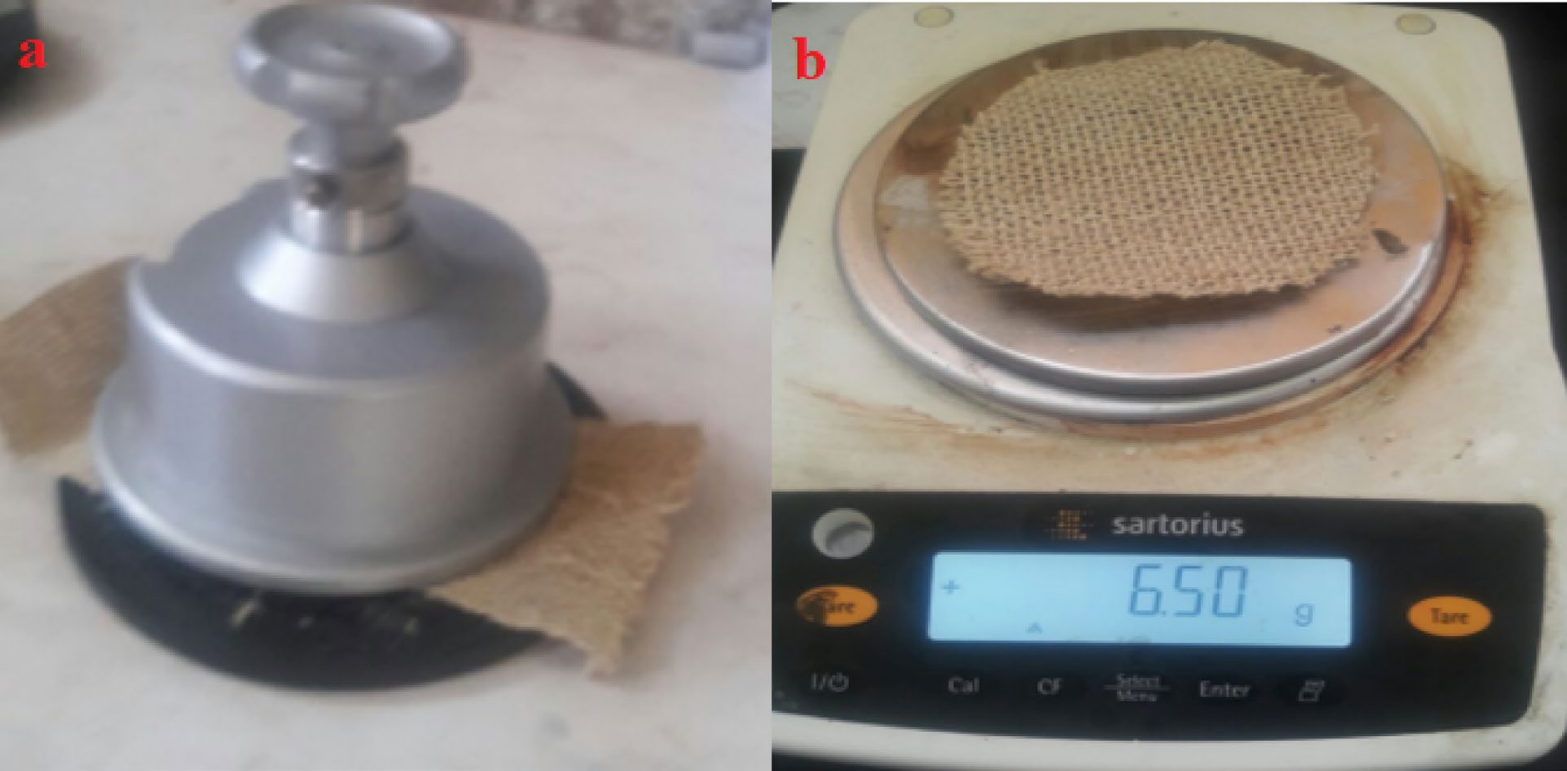
(**a**) GSM cutter test; (**b**) weighing machine.

A GSM cutter was used to calculate the weight of plain mat weave by measuring the mass per unit area. The weight test was performed in accordance with the criteria established by ASTM D3776-96 (2002)^29^ (Fig. 3).Fig. 3(a) GSM cutter test; (b) weighing machine.Fig. 3(a) GSM cutter test; (b) weighing machine.Fig. 3(a) GSM cutter test; (b) weighing machine.

The formula used to determine the weight of the sample is as shown in equation (1):

1 $$GSM=\frac{\text{Weight }\left(\mathrm{gram}\right) \times {10}^{4}}{Area ({cm}^{2})}$$

The average weight of the sample was around 6.50 g per 100 cm^2^. Therefore, the GSM is as shown in equation (2),

2 $$GSM=\frac{6.50\text{ gram }\times {10}^{4}}{100 {cm}^{2}}=650$$

#### Mold preparation

**Fig. 4:**
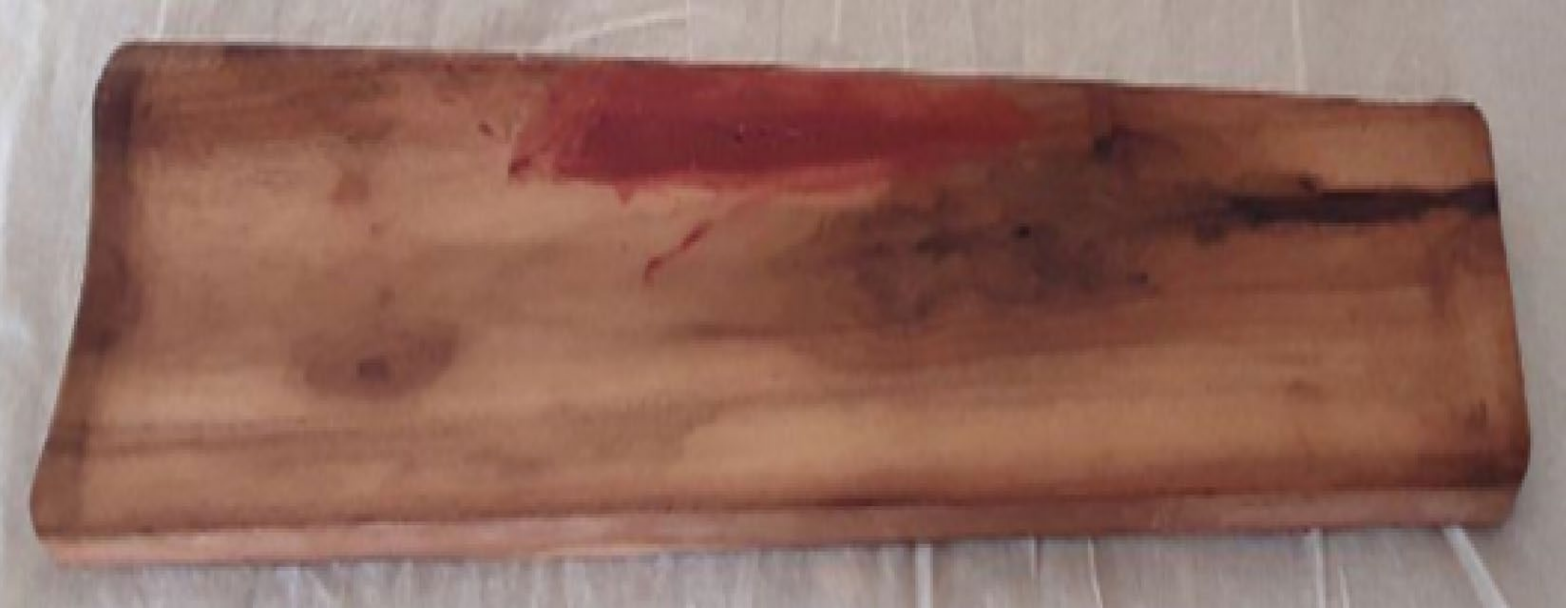
Mold of the ceiling fan blade.

Typically, there are two primary types of ceiling fan blade shapes: aerofoil blades and flat blades. The ceiling fan blade design in this study was inspired by the California Energy Commission’s Center for the Built Environment (CBE)^30^. The selection of the aerofoil blade type was based on the premise that blade curvature enhances airflow through the ceiling fan and reduces air turbulence at the edge, a common issue with flat blades. The mold was fabricated according to the fan blade design specifications (Fig. 4).

#### Experimental tests

The hand lay-up manufacturing technique was employed, and the composite samples were prepared according to proportions determined using a central composite design. After the samples were fabricated based on the software analysis, the physico-mechanical properties, such as water absorption, void fraction, tensile strength, compressive strength, and bending strength, were tested according to the proportional ratios of the factors (fiber and resin).

##### Water absorption

The water absorption of the composite material was evaluated in accordance with ASTM D570-98. Samples with different fiber weight ratios were immersed in a beaker of water at room temperature. The water absorption behavior of the samples was measured after 24 and 48 h of immersion. The water absorption percentage was calculated using Equation (3), as detailed below.

3 $$Water uptake=\frac{Wt-Wo}{Wo}\mathrm{\%}$$

where Wo represents the weight of the composite sample before water immersion (oven-dried weight) in grammes, and Wt denotes the weight of the composite sample after removal from the water in grammes^31^.

##### Density and void fraction

The theoretical density of the material was determined using the weight ratio method to calculate the composite density. The Archimedes method was employed to measure the experimental densities of the composites. According to this principle, when a solid object is immersed in a liquid, the apparent reduction in its weight is equal to the weight of the displaced liquid. This procedure was carried out in accordance with ASTM D792 using the following equation (4):

4 $${\uprho a }=\frac{{\uprho wWa}}{Wa-Ww}$$

where Wa is the weight of the sample in air, Ww is the weight of the sample in water, ρa is the actual density of the composite, and ρw is the density of distilled water. Equation (v) is used to get the volume fraction of voids (5):

5 $$Vvf=\frac{(Pt-Pa)}{Pt}$$

where _“_ρ_t_ represents the theoretical density and ρ_a_ actual density of the composite”^32^.

##### Tensile strength

The tensile strength of the sample was evaluated in accordance with the ASTM E1309 standard, using a composite specimen of dimensions 150 × 20 × 3 mm. The test was performed using a universal testing machine at a crosshead speed of 10 mm/min, and the results were calculated using Equation (6).

6 $$\Delta t=\frac{Wt}{b\times t}$$

where,” δt = Tensile strength (N/mm^2^), Wt = Failure tensile load (N), b = Breadth of the specimen (mm), and t = Thickness of the specimen (mm)”^33^.

##### Compressive strength

The composite samples were tested in accordance with the ASTM D695 standards. Static compressive tests were conducted on specimens measuring 10 mm in height and 10 mm in diameter using a universal testing machine at a crosshead speed of 1 mm/min. Equation (7) was used to calculate the compressive strength.

7 $$\text{Tc }= \frac{\text{Wc }}{b \times t}$$

where “Tc is compressive strength (N/mm^2^), Wc (N) is the failure load, b and t are the breadth and the thickness of the samples in (mm) respectively”^32^.

##### Flexural/bending strength

A three-point bending test was performed on a specimen measuring 80 × 20 × 4 mm in accordance with ASTM D790 standards. The experiments were conducted using a universal testing machine, and the flexural strength of each specimen was calculated using Equation (8).

8 $$\text{FS }= \frac{3\mathrm{PL}}{{2bt}^{2}}$$

where the variables P is the maximum applied force, b is the specimen breadth, t is the specimen thickness, and L is the sample span length^32^.

The formulation containing 30% fiber and 70% resin was selected based on its superior mechanical properties, which facilitated the calculation of Young’s modulus and Poisson’s ratio. These calculations were performed using the epoxy resin density. Consequently, the specified parameters are essential for the finite element analysis (FEA) of the composite materials used in ceiling fan blades. The numerical modeling of the ceiling fan blade began with geometric modeling using the ANSYS Design Modeler module. Subsequently, the geometry was discretized using the ANSYS meshing tool. After these steps, named selections were defined for the geometric boundaries, and the CFD simulation was configured in ANSYS Fluent^34^.

The factors considered in determining the finite element analysis of the ceiling fan blade include rotational speed, geometry, and boundary conditions^35^. A rotational speed corresponding to an airflow velocity of 2 m/s across the blade radius at 200 rpm (21 rad/s) was applied. The geometry was based on standard ceiling fan blade dimensions, and the boundary conditions included three operational constraints: (A) fixed support, (B) rotational velocity, and (C) atmospheric pressure, representing the surrounding room environment of 1 atm (0.101325 MPa). The finite element analysis evaluated blade deformation, von Mises stress, von Mises strain, and airflow behavior.

False banana fiber exhibits superior water absorption and desorption characteristics, attributed to high content of non-cellulosic components, lower crystallinity of the fiber structure, and the presence of hydrophobic chemical constituents such as lignin, lipids, and waxes. Consequently, alkali treatment is required to improve fiber–matrix compatibility and induce physical modifications that enhance mechanical strength^36^. Conversely, unsaturated polyester is a thermosetting resin characterized by excellent crack resistance, flexibility, and chemical stability. Unsaturated polyester resins are widely used in laminates with low volatile organic compound (VOC) emissions. The key properties distinguishing modified unsaturated polyester resins include improved toughness, reinforcement capability, flame retardancy, and enhanced thermal resistance^37^.

## Result and discussion

The physico-mechanical test results of the composite material samples were obtained based on the proportional ratios of fiber and resin using Design-Expert software (central composite design). During the fabrication of the composite materials, the resin and hardener were mixed in a 10:1 ratio and thoroughly blended using mechanical stirring for 15 min.

According to the results presented in Table 2, the optimum physico-mechanical properties were achieved with a composition of 30% fiber and 70% resin. The results indicate a tensile strength of 33.15 MPa, a compressive strength of 29.69 MPa, a bending (flexural) strength of 28.85 MPa, water absorption of 1.54%, and a void fraction of 1.02%.

**Table 2:** Composites with varying proportionate ratios and their physico-mechanical characteristics.

No. of Runs	Factor 1	Factor 2 Response 1		Response 2	Response 3	Response 4	Response 5
	A: Fiber in %	B: Resin in %	Tensile strength in MPa	Compressive strength in MPa	Bending strength in MPa	Water absorption in %	Void fraction in %
1	25	75	33.23	29.56	28.65	1.57	1.02
2	28	72	32.91	28.69	27.82	1.62	1.61
3	20	80	32.86	28.52	27.87	1.55	1.35
4	44	56	32.59	28.46	27.63	2.05	2.01
5	28	72	33.13	29.14	28.14	1.7	1.79
6	30	70	33.15	29.69	28.85	1.54	1.02
7	28	72	33.21	29.62	28.63	1.6	1.4
8	16	84	33.14	28.98	28.14	1.54	1.02
9	45	55	32.61	28.49	27.68	2.02	1.94
10	28	72	32.87	29.13	28.29	1.91	1.9
11	28	72	33.05	29.59	28.62	1.57	1.3
12	40	60	32.82	28.73	28.01	1.91	1.92
13	15	85	32.66	28.35	27.95	1.56	1.25

### Tensile strength

**Fig. 5:**
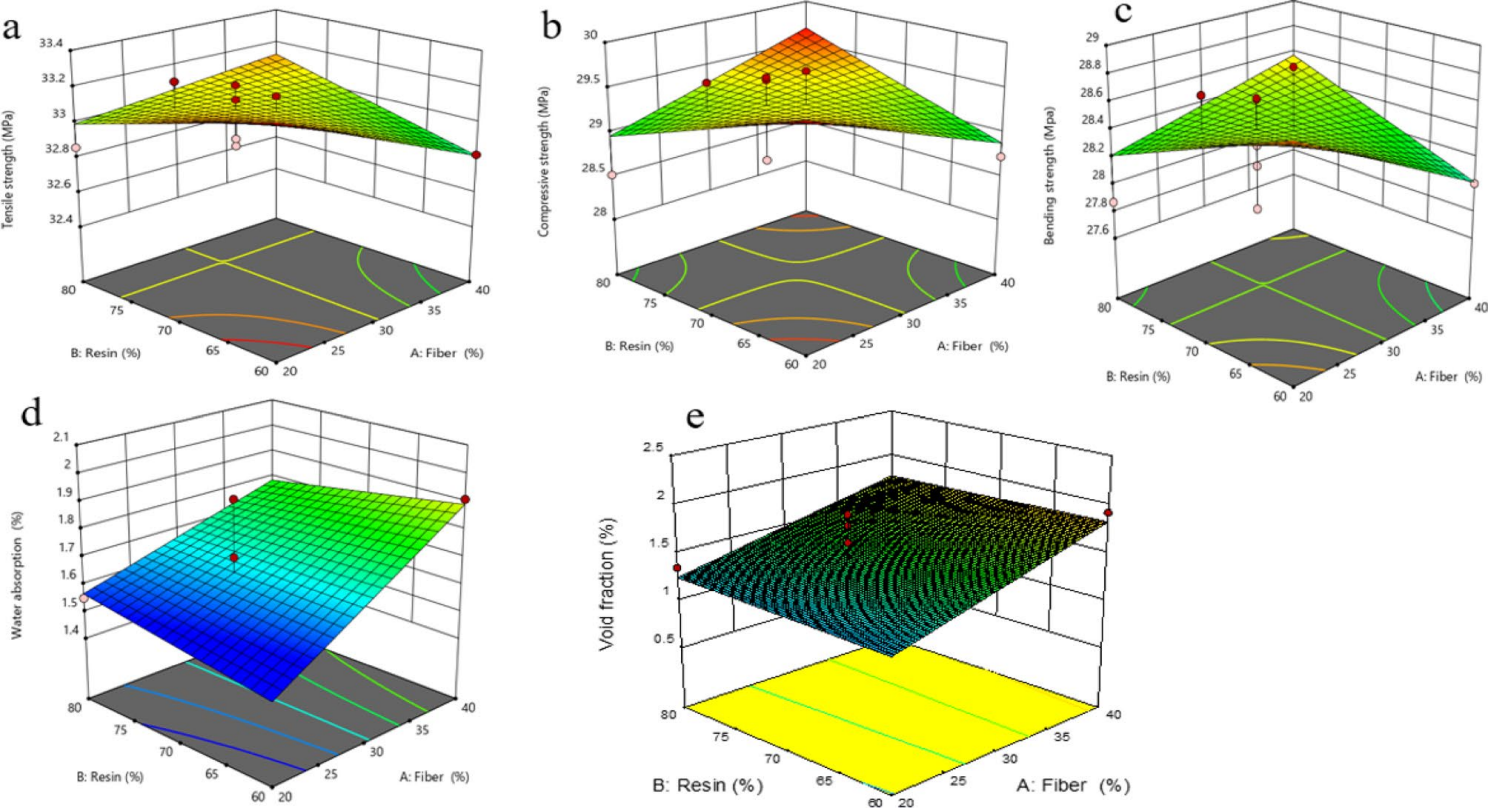
The impact of resin and fibre on (**a**) tensile strength (**b**) compressive strength (**c**) bending strength (**d**) water absorption (**e**) void fraction.

The tensile properties of false banana fiber-reinforced unsaturated polyester composites are influenced by different fiber-to-resin ratios. As shown in Fig. 5, the tensile strength of the composite increases with increasing fiber weight percentage, reaching a maximum of 33.15 MPa at 30% fiber loading. This optimal point represents a balance between sufficient fiber content for load-bearing and adequate resin volume for complete fiber wetting and effective stress transfer at the interface. Beyond this critical fiber content, further increases in fiber loading do not enhance tensile strength, as the matrix becomes insufficient to ensure effective bonding between the fiber and the matrix^38,39^. For instance, at the maximum fiber loading of 44%, the tensile strength was 32.59 MPa, while at the minimum loading of 15%, it was 32.66 MPa^39^. The slight decrease at higher fiber loadings can be attributed to poor fiber wetting and increased void formation. Table 3 shows that the tensile strength of the model was determined using values obtained from statistical linear regression. Establishing the correlation coefficient for both intra-group and inter-group models is essential.

**Table 3:** Analysis of variance for tensile strength.

Source	Sum of Squares	Degree of freedom	Mean Square	F-value	*p*-Value	
Model	18.22	2	9.11	7.67	0.0096	Significant
A-Fiber	6.55	1	6.55	3.40	0.0134	
B-Resin	12.44	1	12.44	3.54	0.0213	
Lack of fit	10.35	9	1.15	1.32	0.0662	Insignificant

The variables were determined to be statistically significant, as the p-value was below 0.05. Consequently, it can be inferred that the parameters influence the tensile strength of the composite material. The p-value represents the significance of the difference between theoretical and observed values. A high p-value indicates a greater probability that the difference is not statistically significant, whereas a low p-value signifies a meaningful difference^40^. Moreover, an increase in the p-value may render the discrepancy between the actual and theoretical values statistically insignificant. Nevertheless, the lack-of-fit F-value is 1.32, with a p-value of 0.0662, indicating that the lack of fit is not statistically significant. This suggests that even when the material is produced with identical fiber-to-resin ratios, test results may vary due to additional factors, such as applied load and environmental conditions^41,42^. As the resin and fiber content increase, the tensile strength correspondingly increases until the peak value is reached.

### Compressive strength

The compressive strength of false banana fiber–reinforced unsaturated polyester composite material was evaluated at various fiber-to-resin ratios. Fig. 5a illustrates that the compressive strength of the composite increases with increasing fiber loading, owing to the uniform distribution of fibers and improved interfacial bonding between the fibers and the matrix, reaching a maximum of 29.69 MPa at 30% fiber loading. This optimum loading facilitates effective stress transfer from the matrix to the fibers. Under these conditions, stress is uniformly distributed among the fibers, resulting in the highest attainable compressive strength^43^. However, when the fiber content exceeds the critical loading level, the compressive strength no longer increases, as the matrix material becomes insufficient to ensure effective bonding between the fibers and the matrix. For example, the compressive strengths were 28.35 MPa at the minimum (15%) fiber loading and 28.46 MPa at the maximum (44%) fiber loading^39^. Table 4 shows that the model’s compressive strength was adjusted based on the values suggested by statistical linear regression. The correlation coefficient of the model should be calculated for both the intra-group and inter-group relationships.

**Table 4:** Analysis variance for compressive strength.

Source	Sum of squares	Degree of freedom	Mean Square	F-value	P-value	
Model	17.12	2	8.56	6.64	0.0146	Significant
A-Fiber	6.27	1	6.27	2.45	0.0262	
B-Resin	10.54	1	10.54	2.17	0.0178	
Lack of fit	8.10	9	0.90	3.56	0.1331	Insignificant

The F-statistic of the model is 6.64, indicating that the model is statistically significant, and the p-value of less than 0.05 suggests that the model components—specifically, the fiber and resin—are also highly significant. These parameters influence the compressive strength of the composite. Nevertheless, the lack-of-fit F-value is 3.56, with a p-value of 0.1331, indicating that the lack of fit is not statistically significant. This suggests that even when the material is produced with identical fiber-to-resin ratios, the test results may vary due to several other factors, including the applied load and ambient environmental conditions.

### Bending strength

The composite material was tested to determine its bending strength using the three-point bending method. The bending strength of the composite reinforced with false banana fiber shows an increase with increasing fiber loading up to an optimum point, as illustrated in Fig. 5c. The bending strength increases with fiber loading up to 30%, reaching 27.95 MPa. The maximum bending strength of 28.85 MPa is achieved at 30% fiber loading, which is attributed to the uniform distribution of fibers and strong interfacial bonding between the fiber and the matrix^44,45^. Beyond this optimum, increased fiber content may lead to poor resin penetration and higher void content, promoting premature failure under bending stress^39^. The model’s bending strength was adjusted based on the recommended value obtained through statistical linear regression, as presented in Table 5. Establishing the correlation coefficient for both intra-group and inter-group models is essential.

**Table 5:** Analysis of variance for bending strength.

Source	Sum of squares	Degree of freedom	Mean Square	F-value	*P*-value	
Model	20.66	2	10.33	9.35	0.0416	Significant
A-Fiber	8.42	1	8.42	3.42	0.0341	
B-Resin	12.22	1	12.22	3.35	0.0318	
Lack of fit	8.82	9	0.98	3.86	0.2143	Insignificant

The model’s F-value of 9.35 indicates statistical significance, and the p-value being below 0.05 confirms that the variables are statistically significant. These factors influence the bending strength of the composite. The lack-of-fit F-value is 3.86, with a p-value of 0.2143, indicating that the lack of fit is not statistically significant. This implies that even when the material is produced with the same fiber-to-resin ratio, test results may vary due to several factors, including the applied load and ambient environmental conditions.

### Water absorption

When the fiber loading increases, the moisture absorption of the composite materials rises rapidly and linearly until the saturation point is reached. A higher percentage of moisture absorption adversely affects the composite material, which in turn deteriorates its mechanical properties^46^. The maximum water absorption was observed at a fiber loading of 44%, as illustrated in Fig. 5d, with a measured absorption rate of 2.05%. Before measuring the weight of each composite sample, the specimens were dried in an oven, and the recorded mass was considered the initial weight of the composites^47^. The samples were then immersed in distilled water maintained at 25 °C (approximately room temperature) for 48 h. The difference in weight before and after immersion was used to determine the amount of water absorbed by the composites. The 48-h immersion test (ASTM D570) provides a standard comparative measure. However, for long-term indoor applications, future work should involve hygrothermal aging studies to model moisture diffusion and assess property retention over time. The statistical linear regression values presented in Table 6 were used as the basis for modeling water absorption.

**Table 6:** Analysis variance for water absorption.

Source	Sum of squares	Degree of freedom	Mean Square	F-value	*P*-value	
Model	33.02	2	16.51	15.99	0.0080	Significant
A-Fiber	5.87	1	5.87	27.03	0.0042	
B-Resin	7.96	1	7.96	29.64	0.0048	
Lack of fit	2.601	9	0.289	27.56	0.9372	Insignificant

The model was determined to have an F-value of 15.99. The p-value is below the threshold of 0.05, which supports the conclusion that the fiber and resin terms in the model are significant, in addition to the overall significance of the model. Furthermore, these parameters influence the bending strength of the composite. However, the p-value of 0.9372 and the lack-of-fit F-value of 27.56 indicate that the lack of fit is not statistically significant. This demonstrates that even when the material is fabricated with the same fiber-to-resin ratio, the test results may vary due to several factors, such as the applied load and ambient environmental conditions.

### Void fraction

Research indicates that the primary deficiency in fiber-reinforced composites is the void fraction, which subsequently affects the mechanical properties of the composite material. The void volume content (Vv) of fiber-reinforced composites is categorized according to ASTM D 792 as follows: less than 0.2% is excellent, less than 0.5% is very good, less than 1% is good, less than 2% is fair, less than 5% is poor, and greater than or equal to 5% is extremely poor^48,49^. The current investigation shows that the void fraction of the fan blade is 1.02%, classified as moderate and within the acceptable limit. As the fiber loading increases from its minimum to maximum value, the void fraction also rises linearly, as illustrated in Fig. 5e. Table 7 demonstrates that the value derived from statistical linear regression was used to compute the model’s void fraction. Establishing the correlation coefficient for both intra-group and inter-group models is essential.

**Table 7:** Analysis of variance for void fraction.

Source	Sum of squares	Degree of freedom	Mean Square	F-value	*P*-value	
Model	22.42	2	11.21	11.11	0.0029	Significant
A-Fiber	4.83	1	4.83	20.22	0.0021	
B-Resin	5.74	1	5.74	22.55	0.0048	
Lack of fit	5.40	9	0.60	7.06	0.8193	Insignificant

The model becomes significant when the F-value is 7.06, and the p-value being below 0.05 further confirms the significance of the variables. These parameters influence the void percentage of the composite material. However, the p-value of 0.81393 and the lack-of-fit F-value of 7.06 indicate that the lack of fit is not statistically significant. This means that for material developed with the same fiber-to-resin ratio, the tested results may vary due to factors such as applied load and ambient conditions (Table 8).

**Table 8:** Comparison of tensile strength and flexural strength of some natural fiber reinforced composites^50^.

S. N	Composite materials constituents	Tensile strength (MPa)	Flexural strength (MPa)
1	Banana fiber + Epoxy	16.20	50.90
2	Banana fiber + Polyester	33.90	50.00
3	Coir fiber + Epoxy	6.69	35.42
4	Sisal fiber + Epoxy	31.00	51.50
5	Jut fiber + Epoxy	42.14	168.74
6	Popular wood dust + HDPE	12.40	27.20
7	False banana fiber + Unsaturated polyester (UP)	33.23	28.85

## Finite element analysis of the ceiling fan blade

Finite element analysis (FEA) is a powerful numerical method for analyzing crack propagation problems**.** The boundary conditions for the FEA were defined by adapting the real-world scenario to conditions suitable for ANSYS software**.** For the finite element analysis of the fan blade, various parameters must be carefully considered and appropriately selected**.**

### Boundary conditions and loads

**Fig. 6:**
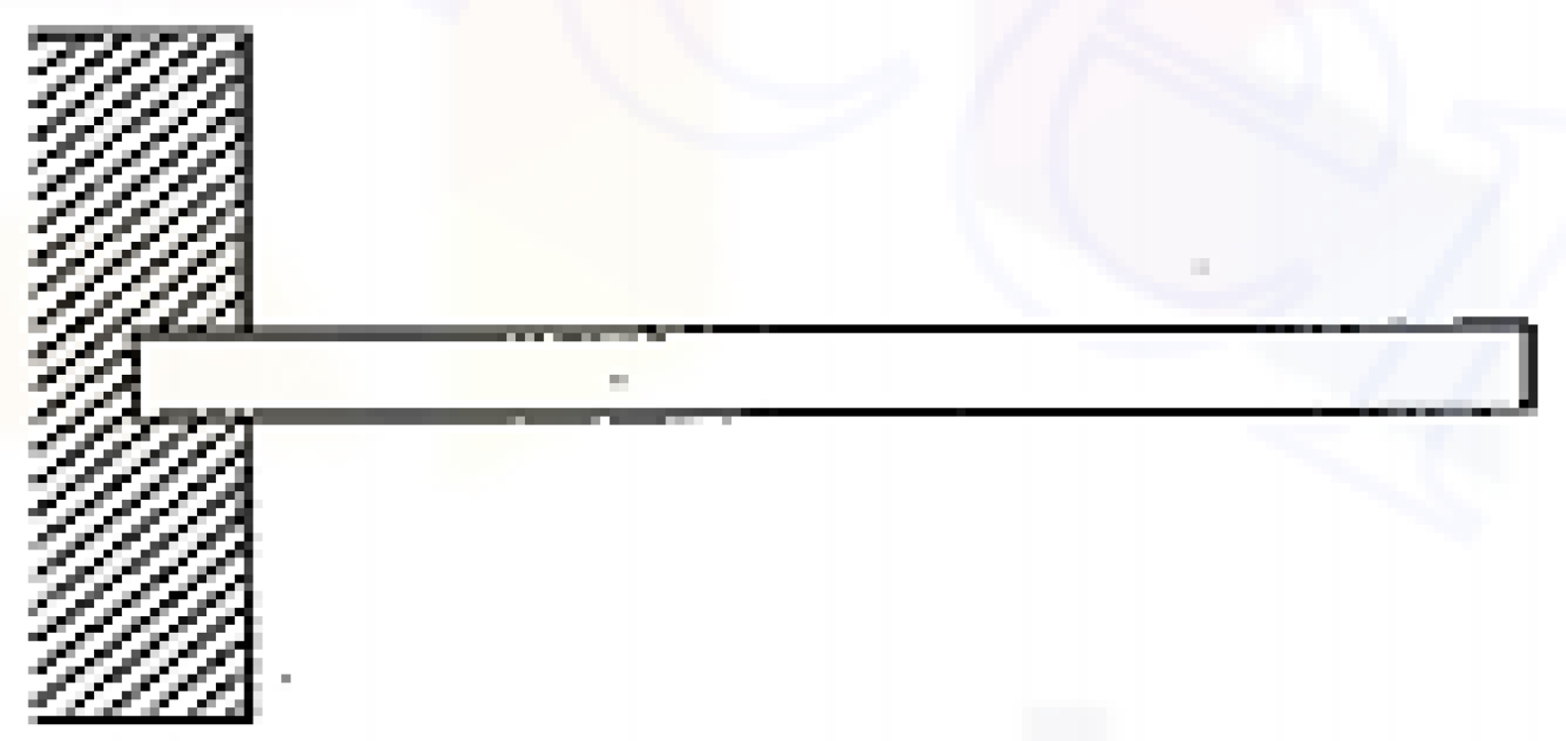
The cantilevered beam^51^.

The boundary condition on the fan blade mirrors that of a cantilever beam, with one end fixed and the other end free. The cantilevered beam is shown in Fig. 6.

**Fig. 7:**
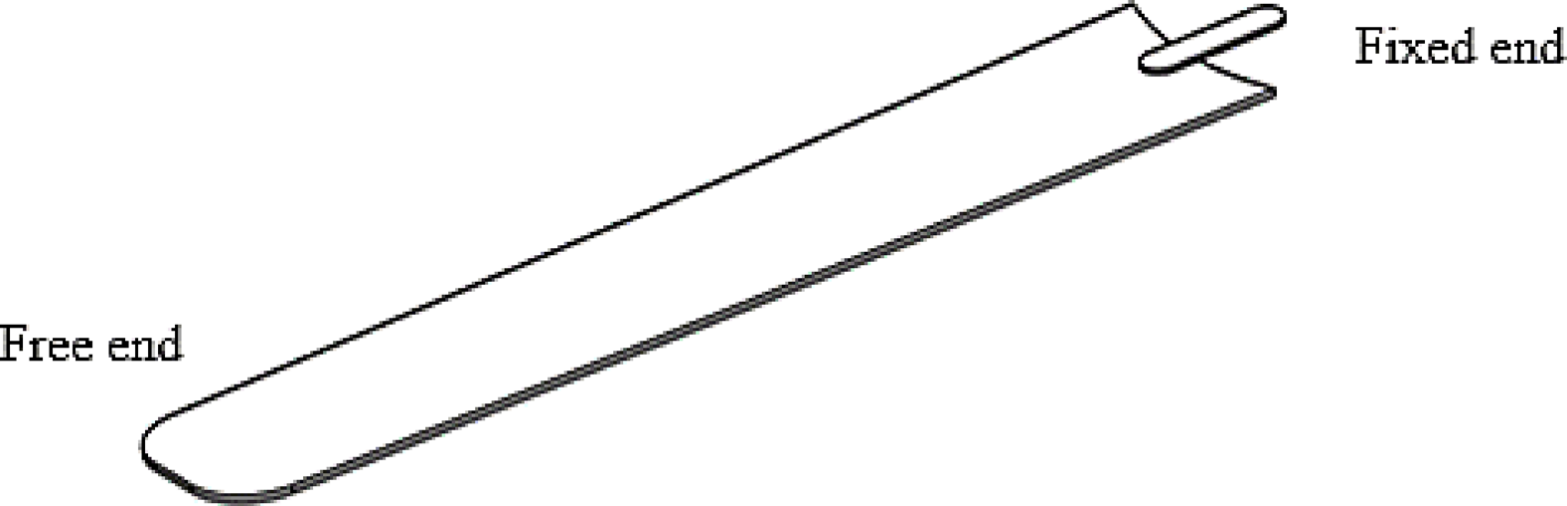
The approximated fan blade (modelled by the researcher in Catia V5).

Here, the fan blade is considered a cantilevered beam since one end is secured to the hub while the other end remains free (Fig. 7).

The primary load considered is the centrifugal force due to rotation. A standard operational speed of 200 RPM (21 rad/s) for residential ceiling fans was selected. The mass of the blade is 0.18 kg, and the radius to the center of mass is 0.6 m. The centrifugal force was calculated,

$$\mathrm{F}=\mathrm{m}{\upomega }^{2}\text{r }(\mathrm{XI})$$

$$F=0.18\, \mathrm{kg}\times {(21\, \mathrm{rad}/\mathrm{s})}^{2}\times 0.6\, \mathrm{m}=47.628\, \mathrm{N}$$

This force was applied as a remote force at the center of mass. Gravitational loads are negligible compared to the centrifugal force at this rotational speed. The boundary condition is a fixed support at the blade root (hub connection). The parameters for the finite element analysis include the beam type, which is a cantilevered beam, the fixed support, and the applied force generated by the speed of the fan blade, as well as the material composition of 70% resin and 30% fiber in the composite. The cross-sectional area at the fixed end measures 120 mm × 3 mm; therefore, the moment of inertia is calculated as follows.

$$I=\frac{{120 \,\mathrm{mm}\times (3\, \mathrm{mm})}^{3}}{12}={270\, \mathrm{mm}}^{4}$$

The half distance is 1.5 mm, and the maximum bending moment at the fixed support is 4820 N-mm. Based on the bending stress formula, let’s calculate the stress.

$${\sigma }_{b}=\frac{MC}{I}=\frac{4820\, \mathrm{N}.\mathrm{mm}\times 1.5\, \mathrm{mm}}{{270\, \mathrm{mm}}^{4}}=26.77\, \mathrm{MPa}$$

### Mesh convergence analysis

**Fig. 8:**
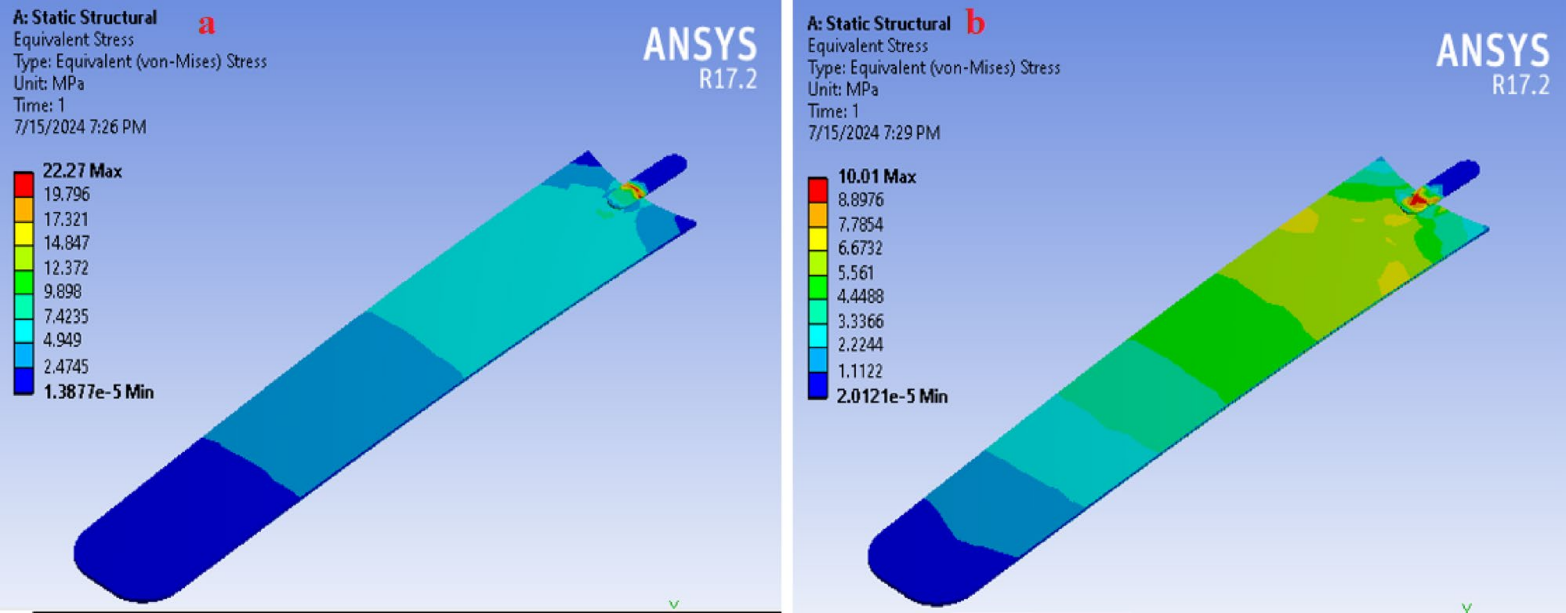
Finite element model of the fan blade showing (**a**) the meshed geometry and (**b**) the applied boundary conditions (fixed support and rotational velocity) (Table 9).

To obtain a suitable mesh with the given parameters, it is necessary to optimize the mesh size to approximate the bending stress to the analytical value of 26.77 MPa. This process is performed manually by adjusting the mesh size and ensuring that the bending stress converges toward the analytical value^52,53^. Some of the results are depicted in the Fig. 8. The mesh size was then varied manually, and the resulting von Mises stress was analyzed and compared with the analytical value**.** The mesh size ranged from 8 mm to 0.25 mm**.**

**Table 9:** Mesh size and the corresponding von Mises stress of the fan blade.

Mesh (mm)	0.25	0.5	1	2	3	4	5	6	7	8
Stress (MPa)	22.27	22.27	22.27	10.01	15.881	13.222	15.329	11.059	12.095	12.713

**Fig. 9:**
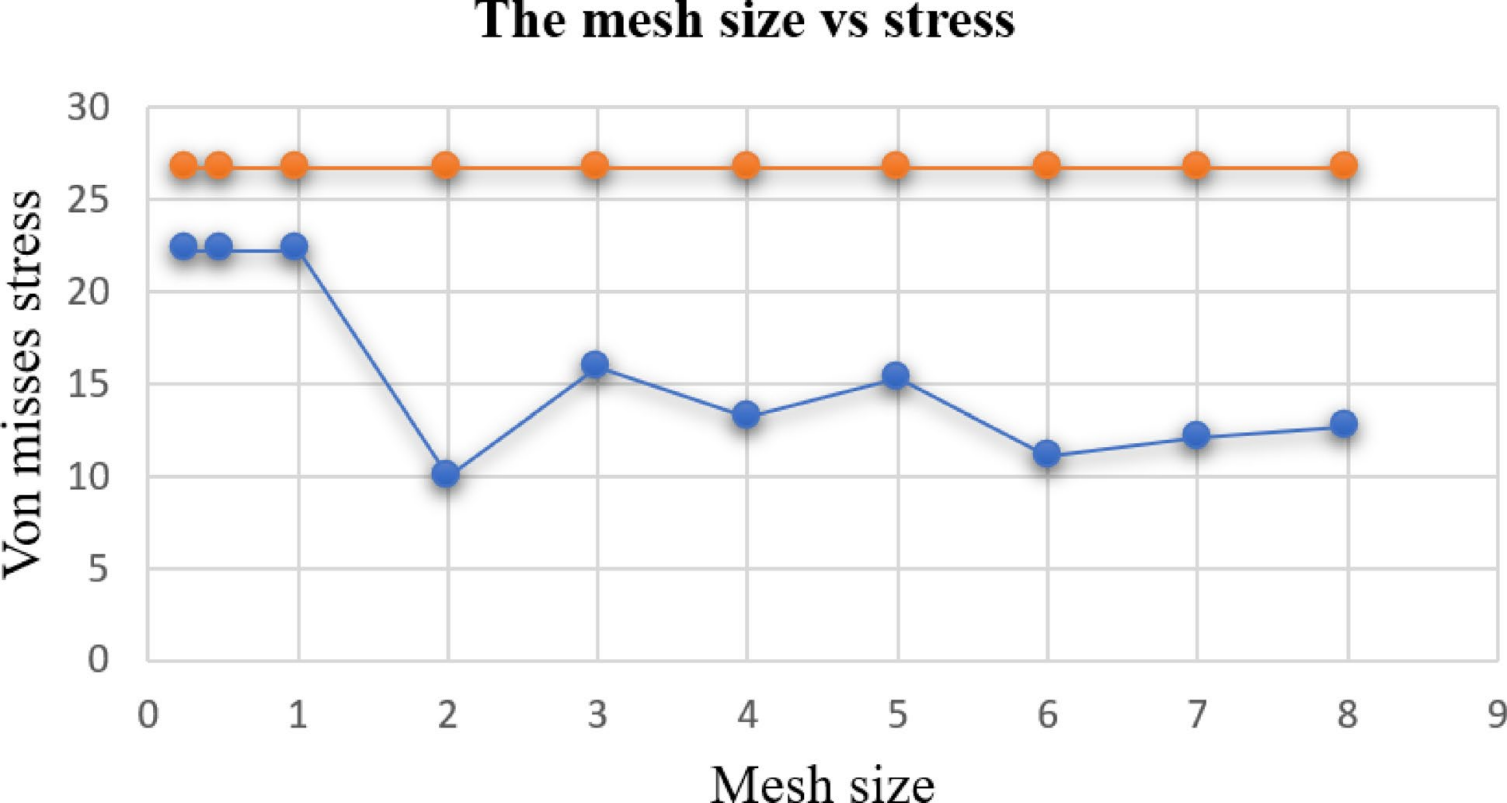
The graph of mesh size and von Mises stress for mesh size selection.

As depicted in Fig. 9, the variation in mesh size from 8 mm to 0.25 mm affects the computed von Mises stress values. At mesh sizes of 0.25 mm, 0.5 mm, and 1 mm, the stress value converges to 22.27 MPa, which is close to the analytical value of 26.77 MPa (Table 9). To optimize computer memory usage during the simulation, a mesh size of 1 mm was selected.

### Convergence and grid independence test of FEM and CFD

At 22.27 MPa, the computed stress varies with mesh size, eventually settling at about 12.71 MPa for coarser meshes, while it reaches 22.27 MPa for the finest mesh (mesh size = 0.25). Although high percentage errors at intermediate mesh sizes (e.g., Mesh Level 4, ~ 54% error) suggest low accuracy with coarser meshes, this demonstrates that the solution converges as the mesh is refined. For the finest meshes (Mesh Levels 1 and 2), the relative error is extremely small, indicating convergence. Around Mesh Level 10, grid independence is achieved, as the percentage error significantly decreases with further mesh refinement. Significant mesh refinement is necessary to reach convergence, with errors dropping below 5% after Mesh Level 8, and the stress readings stabilize. While Mesh Levels 1 to 3 are less efficient due to their high element count (increased computational cost), they produce very accurate results (Table 10).

**Table 10:** Convergence and grid independence test of FEM and CFD.

			Convergence for structural analysis		Convergence for CFD	
Mesh level	Mesh size	Number of elements	Stress (MPa)	% Error	Streamline velocity (m/s)	% Error
1	0.25	921,328	22.27		14.47	
2	0.5	460,664	22.21	0.2694207	14.4	0.48376
3	1	230,332	21.9	1.3957677	14.01	2.708333
4	2	115,166	10.01	54.292237	12.67	9.564597
5	3	57,583	15.881	58.651349	13.21	4.262036
6	4	28,792	13.222	16.743278	8.97	32.0969
7	5	14,396	15.059	13.893511	9.21	2.675585
8	6	7198	11.059	26.562189	8.51	7.600434
9	7	3599	12.095	9.3679356	8.96	5.287897
10	8	1800	12.713	5.1095494	10	11.60714

Grid independence is attained for both studies when variations in the outcomes between subsequent mesh levels are less than a predetermined threshold, such as a percentage error < 1%. The starting velocity is 14.47 m/s for the smallest mesh and varies with increasing mesh size before leveling off at 10 m/s for the coarse meshes. At larger mesh sizes (Mesh Level 10), grid independence is attained, as evidenced by the stability of the streamline velocity. Coarse meshes are unable to adequately capture the flow physics; the inaccuracy is minimal for finer meshes (e.g., 0.48% at Mesh Level 1) but increases significantly for intermediate meshes (e.g., 32% at Mesh Level 6). After Mesh Level 8, the error stabilizes, indicating that the solution is grid-independent and convergent. The results converge and become grid-independent around Mesh Level 10, where the streamline velocity remains constant, while the finest meshes (Mesh Levels 1–3) exhibit modest percentage errors. Significant variations are observed in intermediate meshes (Mesh Levels 4–6), highlighting the importance of using finer meshes to accurately capture aerodynamic phenomena. Grid independence is considered achieved when variations in results between consecutive mesh levels fall below a predetermined threshold, such as a percentage error of less than 1%.

### Deformation

**Fig. 10:**
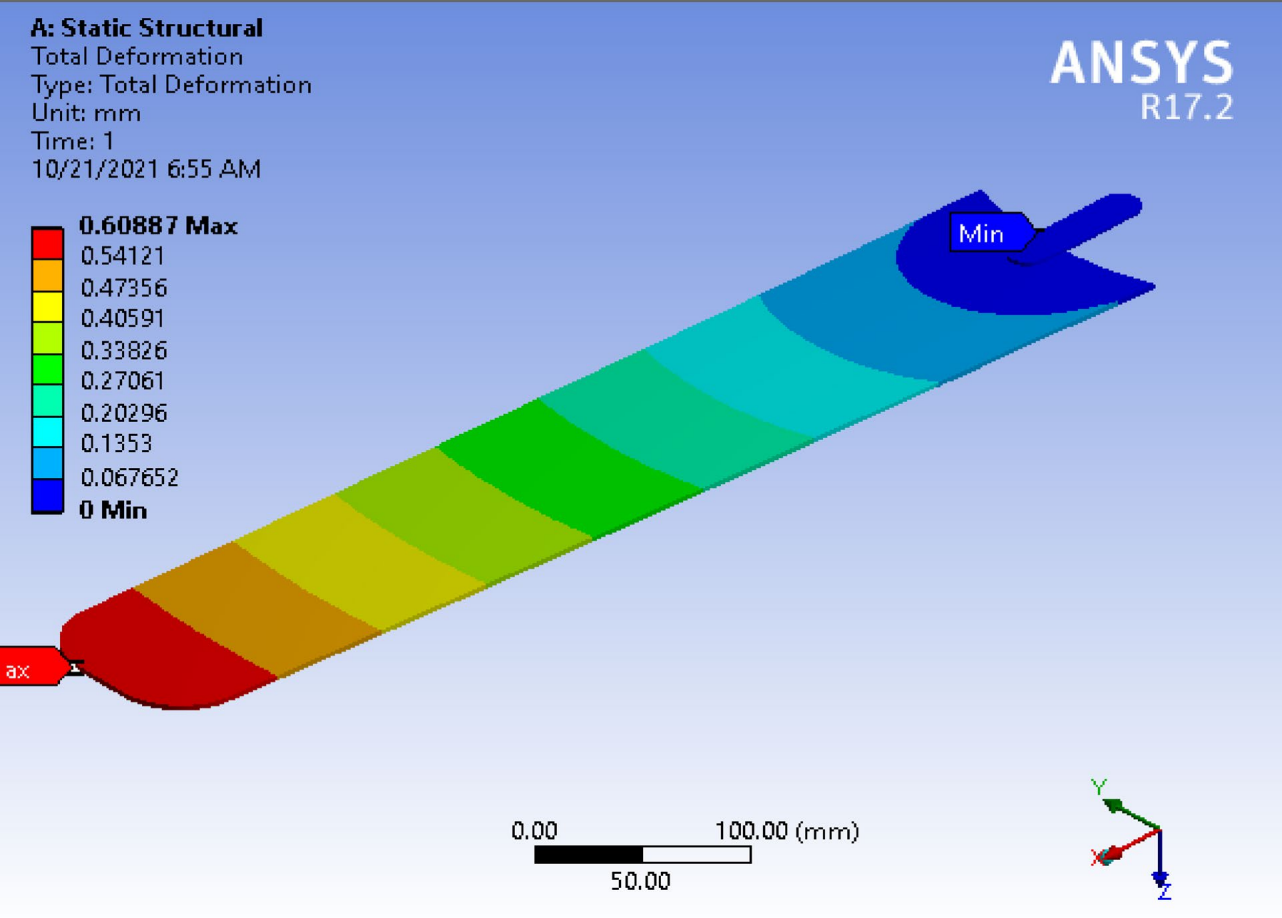
The total deformation of the fan blade.

In materials science, changes in an object’s dimensions or configuration due to applied forces or temperature variations are termed “deformation.” The maximum deformation of the ceiling fan blade occurs near the blade tip, measuring 0.60887 mm. Minimal deformation is also observed at the junction of the blade and the hub, as shown in Fig. 14. Consequently, the overall deformation of the ceiling fan blade is small, indicating that the displacements generated in the blade are minor and within acceptable limits (Fig. 10).

### Von Mises strain and strain energy

**Fig. 11:**
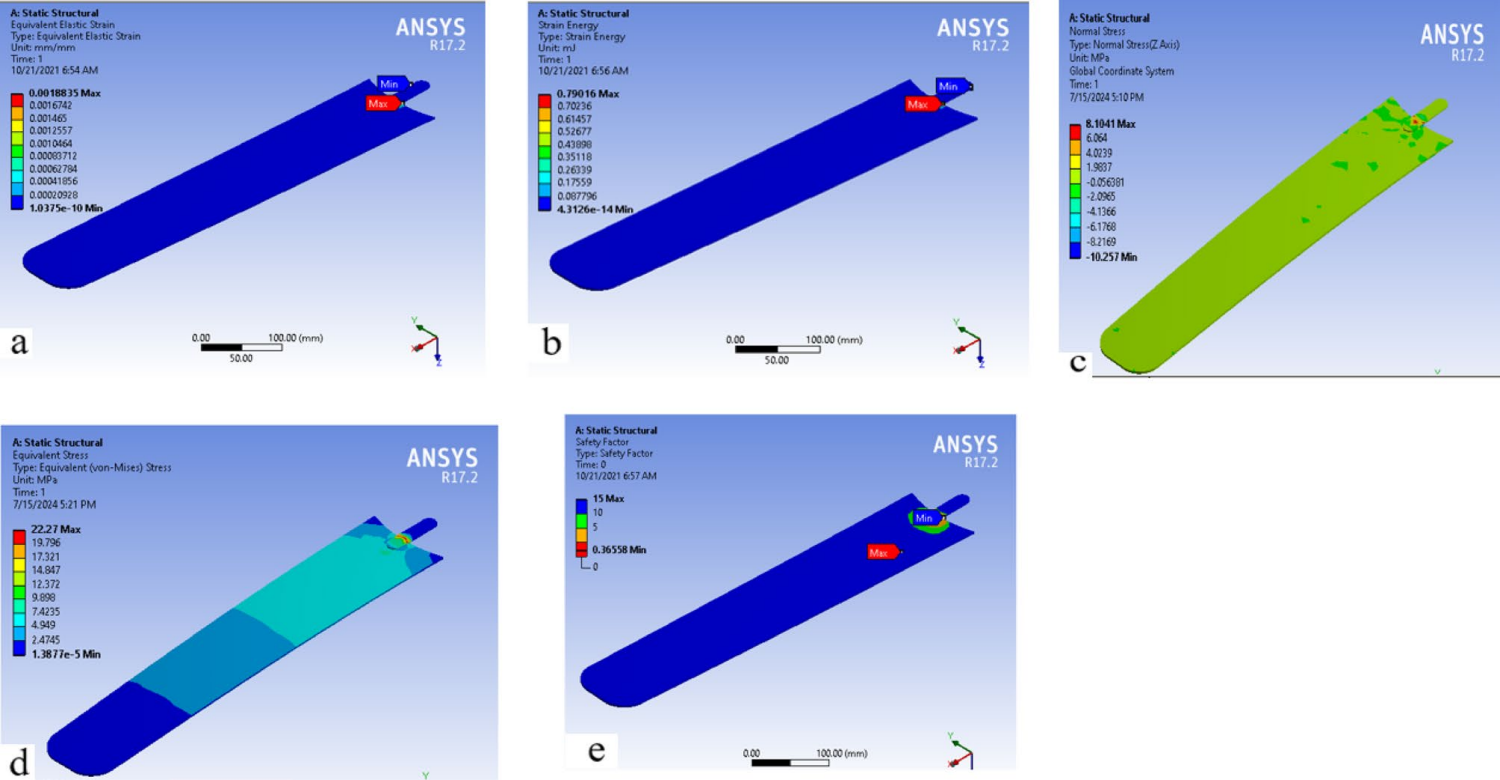
The fan blades (**a**) equivalent elastic strain (**b**) strain energy (**c**) bending stress (**d**) equivalent stress (**e**) factor of safety.

Energy is imparted to a material whenever a load is applied, causing it to deform. This form of energy is referred to as “strain energy.” The preferred approach involves normalizing the strain energy per unit volume, a process known as “strain energy density.” The area under the stress–strain curve represents the energy stored per unit volume; stress multiplied by strain has units of force per area (e.g., N/mm^2^), which is equivalent to N·mm/mm^3^ in terms of energy per unit volume. Only linear elastic materials are considered, and this assumption is generally valid since most metals and alloys exhibit linear elastic behavior before the onset of plastic deformation. Furthermore, Fig. 11a illustrates the distribution of equivalent elastic strain, showing a maximum value of 0.0018835 at the sharp corner, indicated by the red region, and a minimum value of 1.0375 × 10⁻^10^.

Figure 11b shows the strain energy stored in the false banana fiber–reinforced ceiling fan blade, where the maximum value is 0.79016 MJ, observed at the sharp corner of the hub-joining flange, and the minimum value is 4.3126 × 10⁻^14^ MJ. The term “strain energy” refers to the energy stored in a body due to its deformation. The area under the stress–strain curve up to the point of deformation is known as the strain energy density, or strain energy per unit volume. Once the applied force is removed, the system can return to its original configuration, provided the deformation remains within the elastic limit.

### Von mises stress

The von Mises stress criterion is widely used to predict yielding in materials subjected to complex loading conditions by correlating them with results from uniaxial tensile tests. According to this theory, different stress states that produce the same distortion energy will exhibit equivalent von Mises stress values. Figure 11d illustrates the finite element analysis (FEA) results of the equivalent von Mises stress distribution on the composite fan blade. The maximum stress obtained is 22.27 MPa, while the minimum stress is 1.3877 × 10⁻^5^ MPa.

This maximum stress is significantly lower than the tensile (33.15 MPa), compressive (29.69 MPa), and flexural (28.85 MPa) strengths of the composite (Table 2), indicating the blade operates within a safe stress range under the analyzed load. A formal factor of safety specific to the composite’s failure modes would be established in future work using appropriate failure criteria and additional material testing. Furthermore, the deformation pattern observed in the numerical simulation corresponds well with the composite’s measured mechanical behavior, supporting the reliability and accuracy of the FE model.

### Air flow/velocity streamline

A streamline is defined as a line that is tangent to the instantaneous velocity vector. Velocity is a vector quantity, possessing both magnitude and direction. To visualize this concept, one can envision the motion of a defined fluid element. Laminar flow is characterized by a continuous and orderly movement of fluid, such as air, that travels past a solid object while maintaining a uniform direction along its trajectory. The airflow design over the fan blade radius and speed was established based on research conducted by the Florida Solar Energy Center (FSEC) and AeroVironment, Inc.

**Fig. 12:**
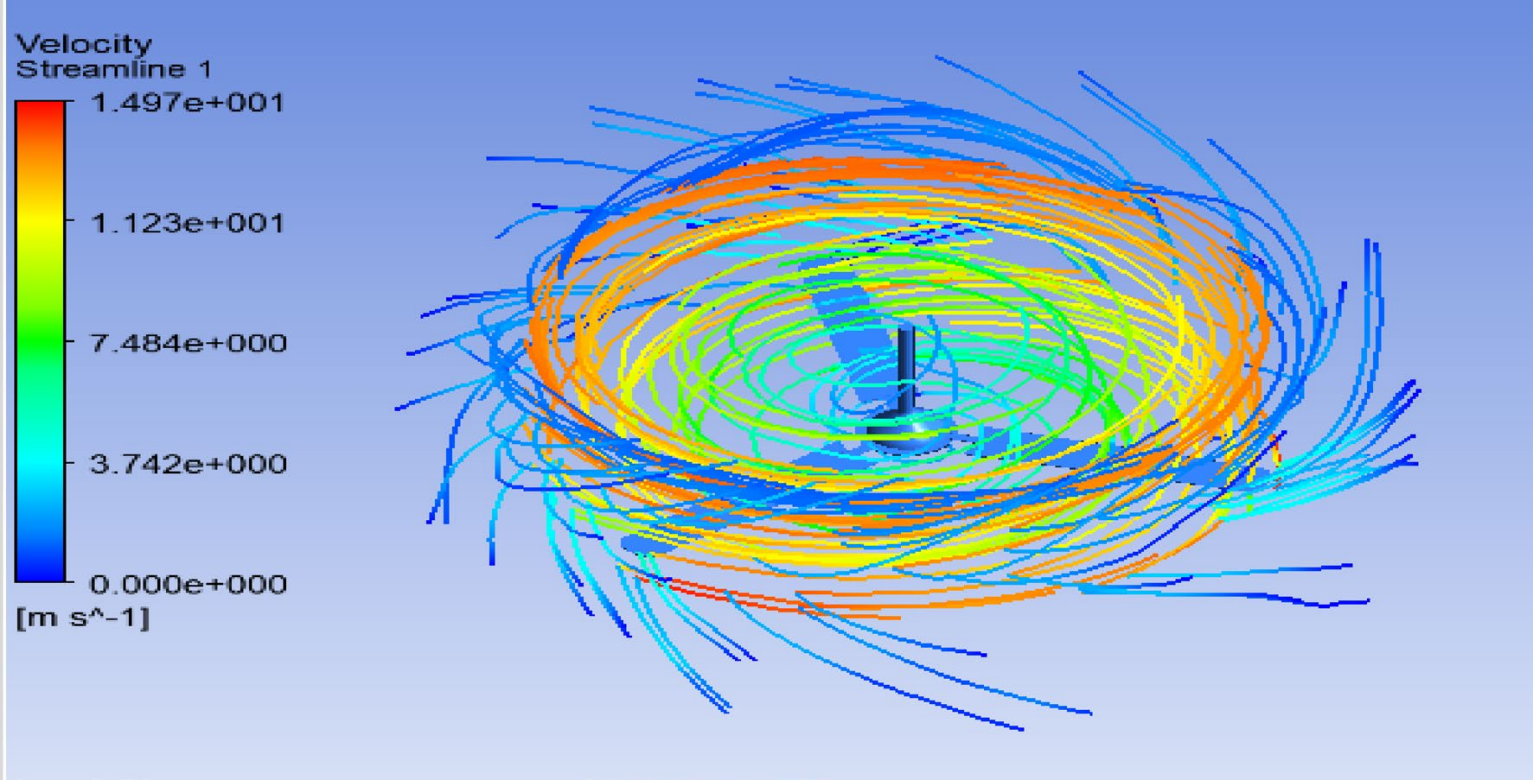
The velocity streamline result.

The speed range was determined to be between 150 and 200 revolutions per minute (rpm), while the fan provides good airflow greater than 0.50 m/s^54^. Hence, as illustrated in Fig. 12, the velocity streamline result of the ceiling fan blade tip in this study is 14.97 m/s, with an input rotational speed of the ceiling fan blade hub of 200 rpm (21 rad/s).

Figure 11e illustrates that the ceiling fan blade exhibits a factor of safety of 15, indicating that the manufactured ceiling fan is structurally safe. Nevertheless, potential failure may occur at the acute angle corresponding to the highest stress concentration. Consequently, this acute angle should be optimized or modified. The analysis demonstrates that the ceiling fan blade is free from significant hazards and does not generate forces that could lead to fracture or structural failure.

### Aerodynamic analysis of the ceiling fan blade

**Fig. 13:**
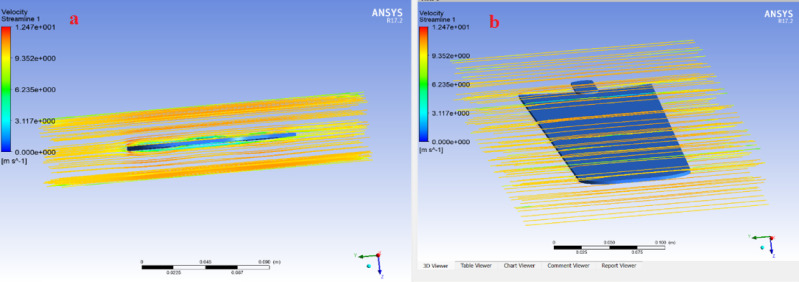
Aerodynamic analysis of the ceiling fan blade: (**a**) velocity magnitude distribution; (**b**) velocity vector direction.

According to Fig. 13, the flow around an object with an aerodynamic profile is illustrated by a velocity streamline plot generated using ANSYS. The velocity streamlines indicate the magnitude and direction of the flow field velocity. The bending of the streamlines around the object demonstrates the interaction between the flow and the geometry, while the smooth yellow streamlines represent undisturbed flow.

The range of velocity values in meters per second is displayed by the color bar. The highest velocity is indicated in red (14.47 m/s), while blue represents the lowest velocity (0 m/s). Due to pressure and boundary layer effects, the flow decelerates near the object (green–blue regions) and accelerates around it (yellow–red regions). The above analysis was carried out using ANSYS Fluent, where the model assesses the aerodynamic properties within a fluid domain, such as lift and drag.

The green and blue regions indicate slower-moving fluid, which may result from boundary layer effects, flow separation, or the object’s influence on the downstream wake. The shape of the object deflects the flow, as evidenced by the streamlines bending around it. Undisturbed flow is indicated by smoother streamlines located farther from the body. The alignment of the streamlines suggests the absence of pronounced turbulence zones in the given image, indicating that the object uniformly influences the surrounding flow field.

## The bending strength of the fan blade

The bending strength analysis of the fan blade under the given loading condition indicates that normal stress is induced in the cross-section relative to the neutral axis (Fig. 11c. The xy-plane is considered the neutral surface, and the y-axis is taken as the bending direction. The maximum bending stress (normal stress along the z-axis) is 8.1041 MPa^55^. This value is compared with the material’s bending strength of 28.85 MPa, as presented in Table 2. Therefore, the material strength is significantly higher than the induced stress, confirming the structural adequacy of the fan blade under bending loads.

## Fatigue analysis of the fan blade

In this section, the fatigue life, alternating von Mises stress, and factor of safety are presented. The safety evaluation of the fan blade also ensures the reliability of the material. This assessment not only presents these critical parameters but also highlights the overall safety of the fan blade material under the specified operating conditions^56^. The fatigue life, which indicates the number of cycles the fan blade can withstand before failure, and the alternating von Mises stress, which characterizes stress variations due to cyclic loading, are key indicators of the blade’s durability. Furthermore, the factor of safety is evaluated to confirm that the blade operates well within safe limits, thereby preserving the structural integrity and reliability of the material^57^.

**Fig. 14:**
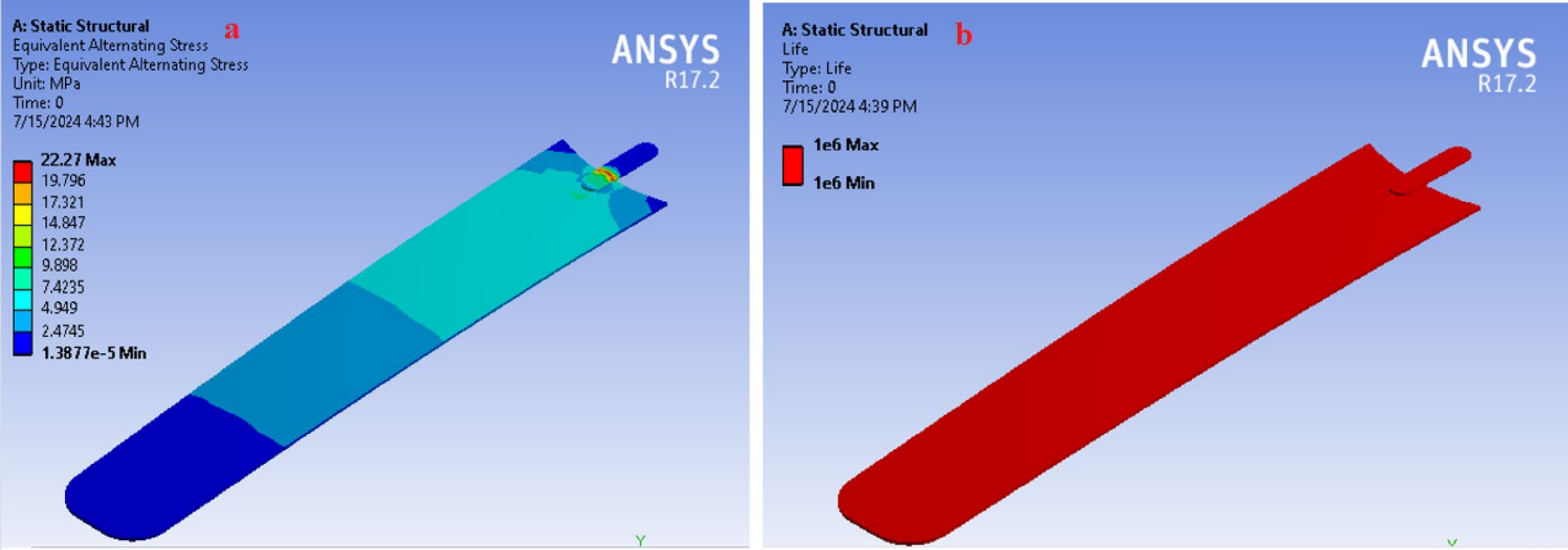
Fatigue analysis of the fan blade showing (**a**) alternating stress distribution and (**b**) predicted fatigue life.

The maximum alternating von Mises stress experienced by the fan blade is 22.27 MPa, which is significantly lower than the material strength values listed in Table 2. These thresholds include a tensile stress of 33.15 MPa, compressive stress of 29.69 MPa, and bending stress of 28.85 MPa^58^. These assessments demonstrate that the maximum stress experienced by the blade is safely within the material limits. Furthermore, the fan blade exhibits a fatigue life of 10⁶ cycles, indicating that it can withstand one million loading cycles before potential fatigue failure. With a factor of safety of 15, this analysis highlights the fan blade’s ability to endure the applied forces and operating conditions with a substantial safety margin, confirming its structural integrity and reliable performance^59^. A comprehensive fatigue life prediction requires the experimental determination of S–N curves for the specific false banana fiber/polyester composite, considering the effects of mean stress and environmental humidity (Fig. 14). Therefore, experimental fatigue testing under representative conditions is identified as a critical requirement for future work to establish the blade’s operational lifespan.

### Additional consideration for fatigue performance

The long-term durability of the composite fan blade under cyclic operational loads is an important factor. A comprehensive fatigue life prediction requires the experimental determination of S–N curves for the specific false banana fiber/polyester composite, considering the effects of mean stress and environmental humidity. Such characterization was beyond the scope of this initial design and fabrication study. Therefore, experimental fatigue testing under representative conditions is identified as a critical requirement for future work to establish the blade’s operational lifespan.

## Comparison of fan blades

**Fig. 15:**
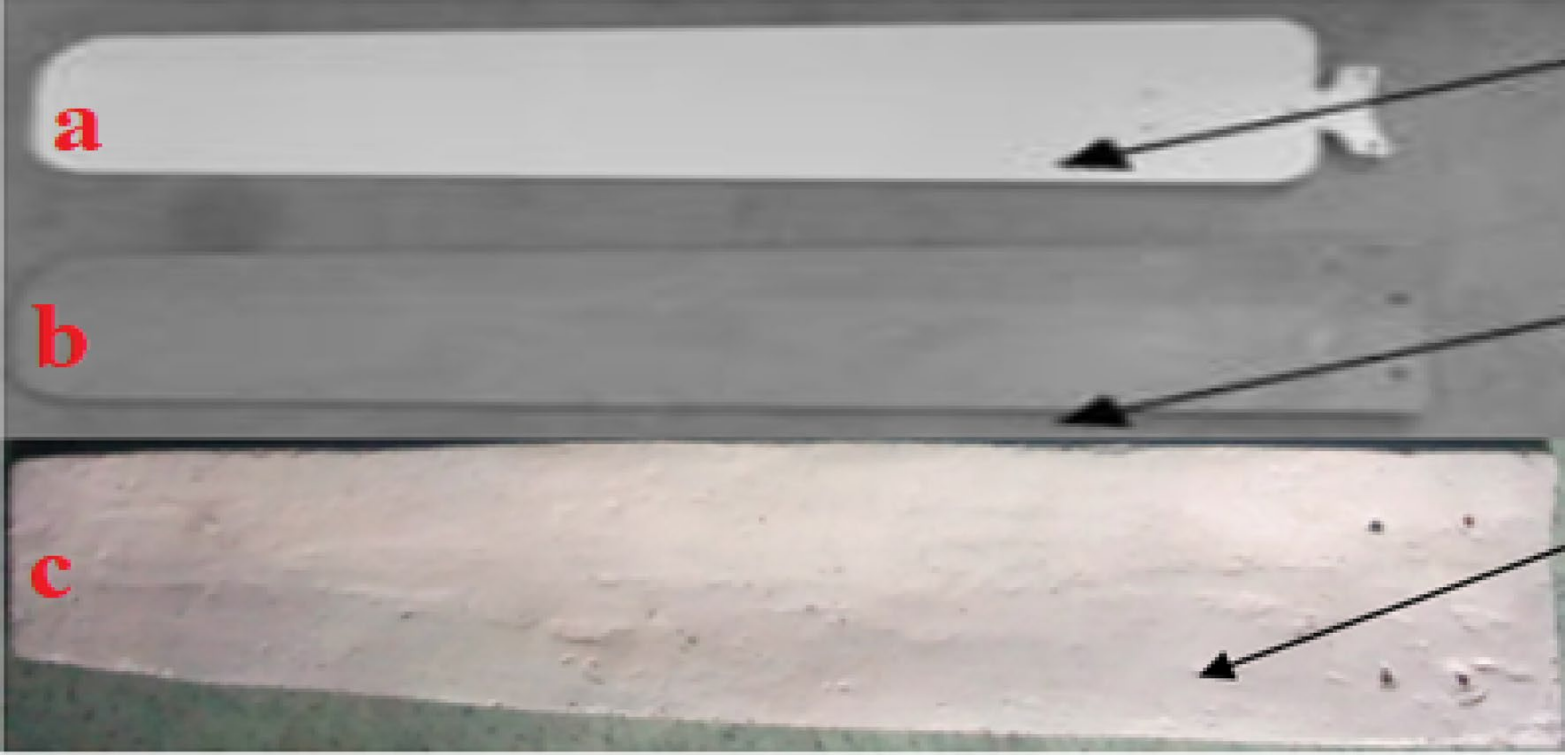
Different types of fan blades (**a**) Aluminum fan blade; (**b**) Glass fiber fan blade; (**c**) False banana fiber fan blade.

The weight of the existing ceiling fan blade is 295 g, while that of the glass fiber–reinforced ceiling fan blade is 215 g, and the false banana fiber–reinforced ceiling fan blade is 205 g^60^, as shown in Fig. 15. The weight reduction is 5% compared with the glass fiber–reinforced ceiling fan blade and 31% compared with the conventional ceiling fan blade. Therefore, the composite material developed using false banana fiber and unsaturated polyester exhibits advantages such as low starting inertia, low cost, good impact absorption, high strength, good energy-saving performance, and high-temperature resistance^61^.

Indeed, the velocity of a ceiling fan blade is intended to produce an air velocity of 2 m/s across the blade radius at a rotational speed of 200 rpm (21 rad/s)^54^. However, the false banana fiber–reinforced ceiling fan blade produced a velocity greater than that reported in the literature, with a measured value of 14.97 m/s, indicating that the streamline velocity is significantly higher than the assumed value. On the other hand, the false banana fiber composite possesses inherent aesthetic properties without requiring surface finishing; however, painting was applied in this case to obtain a uniform surface finish for the composite material.

## Future work

This study demonstrates the feasibility of using false banana fiber-reinforced composites for ceiling fan blades, establishing a foundation based on fundamental physico-mechanical properties and initial design simulation. To advance this work towards commercial application and full structural certification, the following research directions are recommended.

First, a comprehensive characterization of the composite’s long-term and dynamic performance is essential. This includes experimental determination of fatigue life (S–N curves) under cyclic loading, assessment of impact resistance and interlaminar shear strength (ILSS) for rotating components, and evaluation of viscoelastic behavior via dynamic mechanical analysis (DMA). Concurrently, the environmental durability must be established through hygrothermal aging studies to model long-term moisture absorption and its effect on property retention.

Second, the design validation process requires refinement through higher-fidelity modeling and experimental correlation. Future finite element analysis should employ orthotropic material models and composite-specific failure criteria (e.g., Tsai-Wu, Hashin) for accurate failure prediction. These models must be validated through experimental strain gauge measurements on fabricated blades under static and dynamic loads. Furthermore, microstructural analysis using scanning electron microscopy (SEM) is needed to quantitatively assess the fiber-matrix interface quality and understand failure mechanisms, thereby informing further optimization of fiber treatment and composite processing.

## Conclusion

This research focused on developing a composite material for ceiling fan blades, utilizing locally sourced false banana fiber and unsaturated polyester, manufactured through a manual hand lay-up approach. The optimum ratio of reinforcement to matrix in the ceiling fan blade was established based on physico-mechanical properties, utilizing Design Expert software (central composite design) and finite element analysis with ANSYS software. The optimum result was achieved with 30% false banana fiber and 70% unsaturated polyester. The optimal physico-mechanical properties resulted in a tensile strength of 33.15 MPa, a compressive strength of 29.69 MPa, and a flexural strength of 28.85 MPa. Water absorption was 1.54% over 24 to 48 h, and a void fraction of 1.02% was obtained. In addition, finite element analysis revealed a maximum deformation of 0.60887 mm, a maximum equivalent elastic strain of 0.0018835, a minimum strain of 1.0375 × 10⁻^1^⁰, a maximum von Mises stress of 22.27 MPa, a minimum stress of 1.3877 × 10⁻^5^ MPa, and a velocity streamline of 14.97 m/s at 200 RPM (21 rad/s). With a mesh size of 1 mm, the alternating stress on the fan blade was 22.27 MPa, the factor of safety was 15, and the predicted fatigue life of the fan blade was 10⁶ cycles. Furthermore, the false banana fiber-reinforced ceiling fan blade achieved a weight reduction of 5% compared to the glass fiber-reinforced ceiling fan blade and 31% compared to the conventional aluminum ceiling fan blade.

## Data Availability

The authors confirm that the data supporting the findings of this study are available within the article.
